# Myopia Control Effect Is Influenced by Baseline Relative Peripheral Refraction in Children Wearing Defocus Incorporated Multiple Segments (DIMS) Spectacle Lenses

**DOI:** 10.3390/jcm11092294

**Published:** 2022-04-20

**Authors:** Hanyu Zhang, Carly S. Y. Lam, Wing-Chun Tang, Myra Leung, Hua Qi, Paul H. Lee, Chi-Ho To

**Affiliations:** 1 Centre for Myopia Research, School of Optometry, The Hong Kong Polytechnic University, Hong Kong SAR, China; andrea.zhang@cevr.hk (H.Z.); wing922tang@gmail.com (W.-C.T.); chi-ho.to@polyu.edu.hk (C.-H.T.); 2 Centre for Eye and Vision Research (CEVR), Hong Kong SAR, China; 3 Discipline of Optometry and Vision Science, Faculty of Health, University of Canberra, Canberra 2617, Australia; myra.leung@canberra.edu.au; 4 Hoya Corporation, Tokyo 1608347, Japan; hua.qi@hoya.com; 5 Department of Health Sciences, University of Leicester, Leicester LE1 7RH, UK; paul.h.lee@leicester.ac.uk

**Keywords:** myopia, myopia control, myopic defocus, relative peripheral refraction

## Abstract

The aim of this study is to investigate if baseline relative peripheral refraction (RPR) influences the myopia control effects in Chinese myopic children wearing Defocus Incorporated Multiple Segments (DIMS) lenses. Peripheral refraction at 10°, 20°, and 30° nasal (10 N, 20 N, 30 N) and temporal (10 T, 20 T, 30 T) retina were measured at six-month intervals for children who participated in a 2-year randomized controlled trial. The relationship between the baseline peripheral refractions and myopia progression and axial length changes were analysed. A total of 79 children and 81 children in the DIMS and single vision (SV) group were investigated, respectively. In the DIMS group, more baseline myopic RPR spherical equivalent (SE) was associated with more myopic progression (10 N: r = 0.36, *p* = 0.001; 20 N: r = 0.35, *p* = 0.001) and greater axial elongation (10 N: r = −0.34, *p* = 0.001; 20 N: r = −0.29, *p* = 0.006) after adjusting for co-factors. In the SV group, baseline RPR had association with only myopia progression (10 N: r = 0.37, *p* = 0.001; 20 N: r = 0.36, *p* = 0.001; 30 N: r = 0.35, *p* = 0.002) but not with axial elongation after Bonferroni correction (*p* > 0.008). No statistically significant relationship was found between temporal retina and myopia progression or axial elongation in both groups. Children with baseline myopic RPR had statistically significant more myopia progression (mean difference around −0.40 D) and more axial elongation (mean difference 0.15 mm) when compared with the children having baseline hyperopic RPR in the DIMS group but not in the SV group. In conclusion, the baseline RPR profile may not influence future myopia progression or axial elongation for the SV lens wearers. However, DIMS lenses slowed down myopia progression and was better in myopia control for the children with baseline hyperopic RPR than the children with myopic RPR. This may partially explain why myopia control effects vary among myopic children. Customised myopic defocus for individuals may optimise myopia control effects, and further research to determine the optimal dosage, with consideration of peripheral retinal profile, is warranted.

## 1. Introduction

The prevalence of myopia has increased substantially worldwide [1,2] in the last two decades, especially in Asia [3]. High myopia increases the risk of many ocular pathologies, such as glaucoma, retinal detachment, and chorioretinal degeneration, resulting in visual impairment and subsequent deterioration in the quality of daily life [4,5].

Apart from the central foveal area, the peripheral retina also appears to have an important function in emmetropisation [6,7]; animal studies suggest that peripheral myopic defocus can inhibit myopia progression [8], while peripheral hyperopic defocus can induce myopia progression [6,9]. It has been reported that myopic children wearing single vision (SV) spectacles lenses to correct myopia can result in increased hyperopic defocus at the peripheral retina [10], which could promote myopia progression. However, other studies argued that this phenomenon is unlikely to impact myopia progression [11]. Some optical devices applied myopic defocus as a potential way to slow myopia progression in human subjects [12,13,14]. These clinical trials reported that the Defocus Incorporated Soft Contact (DISC) lenses [12], MiSight soft contact lens (Cooper Vision, Inc., Pleasanton, CA, USA) [14], and Defocus Incorporated Multiple Segments (DIMS) spectacle lenses [13] all impose myopic defocus in the central and peripheral retina, with myopia progression being slowed by 50% to 60%. However, the optimal amount of myopic defocus has not yet been determined.

Hyperopic relative peripheral refraction (RPR) has been observed in myopes in cross-sectional studies [15,16,17], while longitudinal studies indicated that changes in RPR, which is typically becoming more hyperopic, might be a consequence of myopia progression rather than a trigger for myopia [18,19,20]. It is not known if baseline RPR influences the result when using DIMS lenses for myopia control. The DIMS lens was designed with a best-corrected zone at the centre and surrounded by multiple segments of constant myopic defocus (+3.50 D) at the mid-periphery, providing clear central vision and peripheral myopic defocus simultaneously [13]. Using real ray tracing and wave optics calculations, we found that viewing a target through the defocus region of the lens leads to ghosting, and the level of ghosting depends on the relative refractive error at the retina [21]. When wearing DIMS lenses, children with lower baseline hyperopic RPR (or with higher myopic RPR) would experience more myopic defocus than children with higher hyperopic RPR. Although the DIMS lens is designed to provide constant myopic defocus of +3.50 D, children might receive a different amount of myopic defocus depending on their actual RPR across the retina. We hypothesised that baseline RPR could influence the effects of myopia control. 

Our previous paper described the characteristics of RPR in Chinese myopic children who participated in a myopia control clinical trial using the DIMS lens [22]. Thus, the current study aims to investigate the link between the baseline RPR and myopia control effects from wearing DIMS lenses and, in this way, provide further insights into optimising myopia control.

## 2. Materials and Methods

### 2.1. Measurements

The children in the current study were participants in a 2-year randomised clinical trial, testing the efficacy of Defocus Incorporated Multiple Segments (DIMS) spectacle lenses for myopia control (Registration Number: NCT02206217) [13]. The recruitment period was from August 2014 to August 2015. All eye examinations and data collection were performed by a registered optometrist at the Centre for Myopia Research at the Hong Kong Polytechnic University. The study was approved by the Human Subject Ethics Sub-committee of the Hong Kong Polytechnic University and adhered to the tenets of the Declaration of Helsinki. Written informed consent was obtained from the parents or guardians of all participating children.

Subject inclusion criteria were [13]:

1. Age at enrolment: 8–13 years
2. Central spherical equivalent refraction (SE): −1.00 to −5.00 D
3. Astigmatism: up to 1.50 D
4. Anisometropia: up to 1.25 D

Exclusion criteria were:

1. Strabismus and binocular vision abnormalities
2. Ocular and systemic abnormalities
3. Prior experience with myopia control

Cycloplegia was induced by one drop of proparacaine (Alcaine 0.5%, Alcon Laboratories, Inc., Fort Worth, TX, USA), followed by 1–2 drops of cyclopentolate HCL 1% (Cyclogyl 1%, Alcon Laboratories, Inc.). Cycloplegic central refraction and peripheral refraction, across the horizontal retina, and corneal power (without cycloplegia) were measured using the Shin-Nippon NVision-K 5001 autorefractor (Ajinomoto Trading Inc., Tokyo, Japan). Central refractive error was measured with the child fixating on a Maltese cross-target placed 3 metres straight ahead [23]. Peripheral refraction was measured at 10°, 20°, and 30° nasally (10 N, 20 N, 30 N) and temporally (10 T, 20 T, 30 T) for the right eye while the left eye was covered. Both central and peripheral refraction were measured without correcting lenses. Axial length (AL) was measured using an IOL Master 500 (Carl Zeiss, Oberkochen, Germany). The standard procedure used for data collection has been described in our previous reports [13].

The spherocylindrical refractions (with the cylinder in negative form) in terms of spherical power (S), cylindrical power (C), and axis (θ) were converted into power vectors using a conventional formula for statistical analysis, namely [24]:

SE = S + C/2
J_0_ = −(C/2) cos (2θ)
J_45_ = −(C/2) sin (2θ)

RPR at a particular eccentricity was calculated by subtracting the central refraction from the respective peripheral refraction. Subjects were subdivided into two subgroups according to baseline RPR: the myopic RPR (RPR ≤ 0 D) and hyperopic RPR (RPR > 0 D) group. Myopia progression and axial elongation over 2 years were further compared between myopic RPR and hyperopic RPR groups within the SV and DIMS group, respectively.

### 2.2. Data Analysis

As there is a high correlation between right and left eyes, only right eye data were analysed [13]. All statistical analyses were performed using IBM SPSS v.16.0 (IBM Corporation, Armonk, NY, USA). The distribution of all data was not significantly different from normal for any of the variables measured (Kolmogorov-Smirnov *p* > 0.05), and data are expressed as mean ± standard deviation (SD). 

The relationships between baseline RPR (independent variable) and myopia progression and axial elongation were determined by multiple linear regression, adjusting for gender, age, and baseline refractive error or AL. Pearson correlation was used to investigate the relationship between baseline RPR, age, myopia progression, and axial elongation. One-way analysis of variance (ANOVA) was conducted to detect the difference in baseline RPR among ages; the Bonferroni post hoc test was applied if necessary. Paired t-tests were used to determine if there were differences in peripheral refraction between the nasal and temporal retina data. An independent t-test was used to compare the difference in myopia progression and axial elongation between children with myopic RPR and hyperopic RPR in the DIMS and SV group, separately. 

As six retinal eccentricities were being considered, a Bonferroni correction was applied, and the significance level was adjusted to 0.008 when analysing parameters related to peripheral refraction.

## 3. Results

Data from 79 children and 81 children in the DIMS and SV group were analysed respectively. There was no statistically significant difference in baseline characteristic data between DIMS and SV groups [13].

### 3.1. RPR SE

At baseline, there was no statistically significant difference in RPR between DIMS and SV groups after Bonferroni correction (*p* > 0.008), and an asymmetrical pattern of RPR profile was found in both the DIMS and SV group.

**Figure 1:**
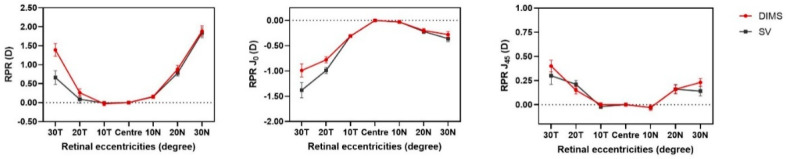
The profile of RPR (SE, J_0_, J_45_) across the horizontal retina of children in DIMS group (n = 79) and SV group (n = 81) at baseline. No statistically significant difference in baseline RPR (SE, J_0_, J_45_) between two groups after Bonferroni correction (*p* > 0.008). Error bars denote SEM. Zero horizontal lines have been shown as dashed lines.

**Figure 2:**
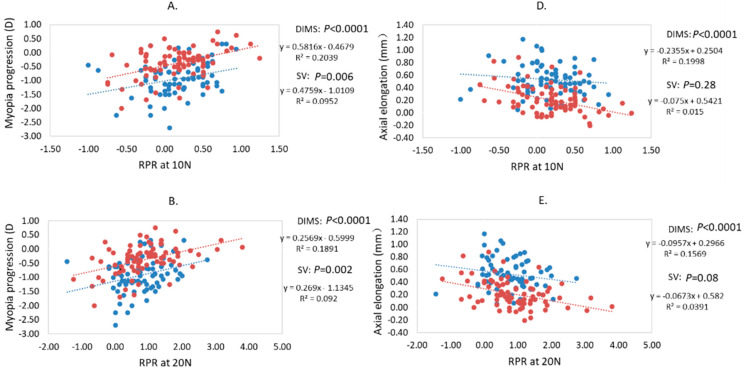

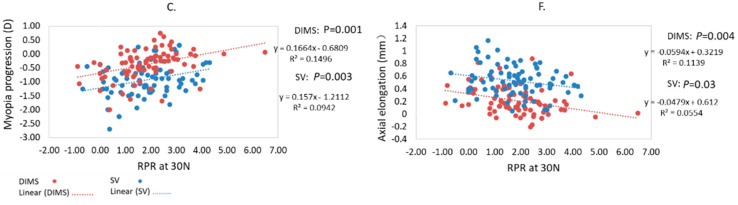
(**A**–**C**): Correlation between baseline RPR, at 10 N, 20 N, 30 N, and myopia progression in the DIMS and SV group over 2 years. (**D**–**F**): Correlation between baseline RPR, at 10 N, 20 N, 30 N, and axial elongation in the DIMS and SV group over 2 years.

Hyperopic RPR SE was observed at most eccentricities across the horizontal retina, except at 10 T in both the DIMS (mean −0.03 ± 0.47 D) and SV group (mean −0.01 ± 0.35 D) (Figure 1). A broad range of hyperopic RPR SE was present at 30 N, ranging from 0.00 to 6.50 D (Figure 2). In the DIMS group, there was asymmetry in RPR SE between the temporal and nasal retina with a more hyperopic RPR SE at 10 N (mean difference: −0.19 ± 0.74, *p* = 0.03), 20 N (mean difference: 0.62 ± 1.36 D, *p* < 0.0001), and 30 N (mean difference: 0.61 ± 1.53 D, *p* = 0.007) compared with the temporal retina. Similarly, in the SV group, there was more hyperopic RPR SE at 10 N (mean difference: −0.15 ± 0.51, *p* = 0.009), 20 N (mean difference: 0.67 ± 1.06 D, *p* < 0.0001), and 30 N (mean difference: 1.09 ± 1.81 D, *p* < 0.001) than in the temporal retina. Only 20 N and 30 N showed a difference that reached a statistically significant level after Bonferroni correction (*p* < 0.008) in both groups.

### 3.2. RPR J_0_, J_45_

Relative astigmatism showed no statistically significant difference between the DIMS and SV groups at baseline. 

Relative J_0_ and relative J_45_ in both DIMS and SV groups showed a similar profile at baseline, without a statistically significant difference after Bonferroni correction (*p* > 0.008, Figure 1). Both relative J_0_ and relative J_45_ increased in magnitude with increasing eccentricity, and the change in magnitude of relative J_45_ was less than relative J_0_. There were no significant differences in relative J_45_ between the nasal and temporal retina in both groups (*p* > 0.05). An asymmetrical profile between the temporal and nasal retina was also found in relative J_0_, with relative J_0_ being more negative at the temporal retina than at the nasal retina in the DIMS group (10° mean difference: −0.28 ± 0.39 D, *p* < 0.0001; 20° mean difference: −0.59 ± 0.75 D, *p* < 0.0001; 30° mean difference: −0.94 ± 0.74 D, *p* < 0.0001) and SV group (10° mean difference: −0.28 ± 0.34 D, *p* < 0.0001; 20° mean difference: −0.77 ± 0.66 D, *p* < 0.0001; 30° mean difference: −1.22 ± 1.06 D, *p* < 0.0001).

### 3.3. RPR and Age

There was no statistically significant difference in baseline RPR among different ages in the SV group and children older than 8 years in the DIMS group (ANOVA, *p* > 0.05). In the DIMS group, only the 8-year-old group had statistically significantly more myopic RPR than the 11-year-old group (Bonferroni post hoc test, mean difference: −0.65 ± 0.16 D, *p* = 0.002). 

**Figure 3:**
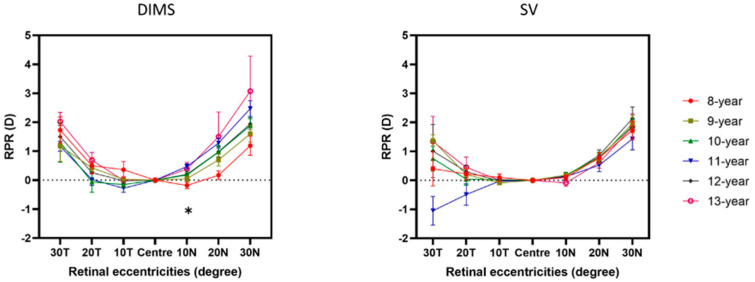
Baseline RPR among each age subgroup in the DIMS and SV group, respectively. * Indicated the significant difference in RPR among age subgroups after Bonferroni correction (ANOVA, p < 0.008). Error bars denote SEM. Zero horizontal lines have been shown as dashed lines.

The RPR profile among each age group is shown in Figure 3. Within each age group, there was no statistically significant difference in baseline RPR between DIMS and SV group after Bonferroni correction (Independent *t*-test, *p* > 0.008).

### 3.4. Relationship between Baseline RPR and Myopia Progression and Axial Elongation

RPR at nasal retina showed a statistical association with myopia progression for both DIMS and SV groups, and statistical association with axial elongation in only the DIMS group (*p* < 0.0008 after Bonferroni correction) (Table 1, Figure 2). There was no statistically significant association of either relative J_0_ or J_45_ with myopia progression or axial elongation in both the DIMS and SV group (*p* > 0.05).

In the DIMS group, baseline RPR SE at 10 N (multilinear regression, r = 0.36, *p* = 0.001) and 20 N (r = 0.35, *p* = 0.001) were positively associated with myopia progression (more baseline myopic RPR, more myopia progression), after adjusting for the co-factors of age, gender, and initial refractive error, and reached a statistically significant level after Bonferroni correction (Table 1). Baseline RPR was negatively associated with axial elongation (10 N, r = −0.35, *p* = 0.001; 20 N, r = −0.30, *p* = 0.004), after adjusting for the co-factors of age, gender, and initial axial length, and reached a significant level after Bonferroni correction (Table 1). 

In the SV group, baseline RPR SE at 10 N (r = 0.37, *p* = 0.001), 20 N (r = 0.36, *p* = 0.001), and 30 N (r = 0.35, *p* = 0.002) were positively associated with myopia progression after adjusted for co-factors (Table 1), but no statistically significant relationship between RPR and axial elongation was found after Bonferroni correction (*p* > 0.008) (Table 1).

The correlation between RPR at the nasal retina and myopia progression and between RPR at the nasal retina and axial elongation in the DIMS and SV groups are illustrated, respectively, in Figure 2.

### 3.5. Comparison between Baseline Myopic RPR and Hyperopic RPR at 10 N and 20 N Subgroups

Only the comparison in myopic RPR (10 N, 20 N) and hyperopic RPR (10 N, 20 N) was presented because other positions did not show a statistically significant correlation with myopia progression and axial elongation. 

In the SV group, there were no statistically significant differences in myopia progression (mean difference: −0.26 ± 0.14 D, *p* = 0.06) and axial elongation (mean difference: 0.04 ± 0.05 mm, *p* = 0.48) between the myopic RPR (n = 27) and hyperopic RPR (n = 54) groups at 10 N (Table 2). There was also no significant difference in myopia progression (mean difference: −0.25 ± 0.20 D, *p* = 0.19) and axial elongation (mean difference: 0.08 ± 0.08 mm, *p* = 0.27) between myopic RPR (n = 11) and hyperopic RPR (n = 70) groups at 20 N (Table 3).

On the contrary, in the DIMS group, myopic RPR at the 10 N subgroup (n = 27) showed statistically significantly more myopia progression (mean difference: −0.36 ± 0.14 D, *p* = 0.009) and axial elongation (mean difference: 0.16 ± 0.05 mm, *p* = 0.001) than the hyperopic RPR at the 10 N subgroup (n = 52) (Table 2). Meanwhile, myopic RPR at the 20 N subgroup (n = 12) showed statistically significantly more myopia progression (mean difference: −0.40 ± 0.16 D, *p* = 0.01) and axial elongation (mean difference: 0.15 ± 0.07 mm, *p* = 0.02) than the hyperopic RPR at the 20 N subgroup (n = 67) (Table 3).

In summary, there was a statistically significant difference in myopia progression and axial elongation between children with baseline myopic RPR and hyperopic RPR in the DIMS group but not in the SV group.

## 4. Discussion

This study investigated baseline RPR and its influence on myopia control effect using the DIMS spectacle lens; this lens is designed to provide simultaneous vision correction and myopic defocus in the mid-periphery of the retina. In the 2 years, RCT has shown an efficacy of 52% in myopia retardation and 62% in less axial length growth [13]. A number of visual functions were shown not to be affected by wearing the DIMS lens [21]. The current study focused on peripheral refraction and how DIMS lens wear affects the change in peripheral refraction. Only horizontal peripheral refraction was measured, as previous studies found no significant association between myopia and peripheral refraction along the vertical meridian [16,25]. 

Our results suggest that, using the DIMS lens for myopia control, children with baseline hyperopic RPR showed less myopia progression and less axial elongation than children with baseline myopic RPR, which suggests that children with hyperopic RPR showed a more effective treatment effect.

### 4.1. RPR in Young Children

Consistent with previous studies, there were no significant differences in baseline RPR among the ages in the SV group and children older than 8 years in the DIMS group [26]. Interestingly, 8-year-old children showed more myopic RPR at nasal retina compared with other ages in the DIMS group (Figure 3). 

### 4.2. RPR in Relation with Myopia Progression and Axial Elongation

Hyperopic RPR SE was observed at most eccentricities among myopic children and increased with eccentricity; these findings were consistent with the results from previous studies [15,16,25]. The RPR profile in myopic children was asymmetric, with more hyperopic RPR at the nasal retina than the temporal retina, and such asymmetry was also reported by previous studies [18,22]. The asymmetry pattern has been suggested to be related to asymmetries in vitreous chamber depth [9] or corneal curvature [25]. 

Although the association between baseline RPR, at the nasal retina, and myopia progression reached a statistically significant level after Bonferroni correction (*p* < 0.008), baseline RPR at the nasal retina only influenced less than 10% of myopia progression variation among the SV wearers (R2 < 0.10). The baseline RPR was not associated with axial elongation in the SV group. Similar results have also been reported by previous studies [20,27]. Atchison [27] followed a group of emmetropic, hyperopic, and myopic children, and found that, although myopes with myopic RPR at baseline were associated with more myopia progression, the emmetropes with myopic RPR at baseline remained emmetropic after the study [20,27]. They suggested that there was a shift from myopic RPR to hyperopic RPR when myopia developed together with eyeball stretching. RPR may not be a trigger of myopia progression [28,29] but a consequence of myopia development or progression, as the eyeball becomes more prolate during axial elongation [25]. Similarly, we also observed hyperopic shifts from myopic RPR to hyperopic RPR in the SV group over 2 years in our previous paper [22].

We further divided the children, according to their baseline RPR, into myopic RPR and hyperopic RPR subgroups. In the SV group, there was no statistically significant difference in myopia progression and axial elongation between myopic RPR and hyperopic RPR at 10 N and 20 N subgroups. Such findings indicate that, whether the baseline RPR profile was myopic or hyperopic, it may not influence future myopia progression or axial elongation for the SV lens wearers.

### 4.3. RPR in Myopia Control Using Myopic Defocus

Our previous studies suggested that, inducing peripheral myopic defocus while simultaneously maintaining clear central vision, such as with the DISC contact lenses [12] or the DIMS spectacle lenses [13], would result in significant myopia control effects. The mechanism was based on the signals of the blur images generated by the myopic defocus received by the retina. This takes into account other factors such as the lag of accommodation [30]. Several studies have demonstrated a reasonable myopic control effect with myopic defocus power ranging from +1.25 D to +3.5 D [12,14,31,32]. If the retinal shape varies at different eccentricities, would the resultant myopic defocus power be different at different eccentric retinal positions? Furthermore, would the baseline RPR profile influence the effects of myopia control? 

It is possible that the initial RPR profile, when superimposed with myopic defocus (+3.50 D) from the DIMS lens, would have a different summation of defocus perceived by the eye at different locations of the retina. For the children with hyperopic RPR at the mid-periphery retina, the defocus power would counterbalance the existing hyperopic RPR. Therefore, less than +3.50 D myopic defocus would be perceived by the retina. However, for the children with myopic RPR, the existing myopic RPR combined with myopic defocus from the DIMS lens would lead to more than +3.50 D of myopic defocus. In the subgroup analysis, we found that children with baseline myopic RPR showed significantly more myopia progression and axial elongation than children with baseline hyperopic RPR. 

The mean myopia progression was −0.72 ± 0.64 D and the mean axial length growth was 0.34 ± 0.24 mm in the children with myopic RPR at 20 N in the DIMS lens group, and was −0.31 ± 0.48 D and 0.19 ± 0.20 mm in the children with hyperopic RPR at 20 N in the DIMS lens group, over 2 years. There was statistically significantly more myopia progression (mean difference −0.40 D) and more axial elongation (mean difference 0.15 mm) in the myopic RPR group than in the children with baseline hyperopic RPR. (Table 3)

When compared with the SV group in the RCT (n = 81) who had no myopia control treatment, their mean myopia progression was −0.93 ± 0.06 D and mean axial length growth was 0.53 ± 0.03 mm [13]; it is apparent that children wearing the DIMS lens benefitted with less myopia progression, but the effect was more pronounced in children with hyperopic RPR. 

The results showed that children with myopic RPR had less effect on myopia control than children with hyperopic RPR, when receiving the additional myopic defocus exposure from the DIMS lens. In fact, in the DIMS group, 8- and 9-year-old children showed more baseline myopic RPR than the older age groups, and they showed less myopia control effects compared with the other age group who had baseline hyperopic RPR [13]. We have reported previously that the effects of myopia control with DIMS lenses have no association with a lag of accommodation, initial myopia, or parental myopia [13]. The possible explanation for the variation of the effectiveness of myopia control was due to the fact that the actual amount of myopic defocus, from the DIMS lenses, received by the eye was influenced by the initial RPR profile. Our findings pointed out that children with baseline myopic RPR might not benefit as much as the children with baseline hyperopic RPR when using DIMS lenses. Without the myopic defocus interference, as in the DIMS group, there was no age variation of myopia progression in the SV group. 

### 4.4. The Effective Range of Myopic Defocus in Myopia Control

The range of adequate defocus power to manipulate refractive error varies between animals, such as between −10 and +20 D in chicks [33], −30 to +5 D in mice [34], and −2 to +8 D in monkeys [35]. There is a decreasing range of defocus for eye compensation from mice, avian, to primate; thus, we assume that humans may have a narrow range of defocus power where eye growth can be manipulated. Garcia et al. [36] reported in a study that superimposing myopic defocus for compensating hyperopic RPR in myopes, in some cases, could degrade the peripheral image quality. DIMS lens wearers with baseline myopic RPR received too much myopic defocus at the mid-periphery retina, which can result in an overall peripheral image blur beyond the threshold of signal detection, and myopia control would therefore be less effective [37,38].

Berntsen et al. [39] studied whether peripheral defocus was associated with myopia progression and suggested that, although peripheral myopic defocus was associated with significantly less myopia progression, a higher amount of peripheral myopic defocus did not slow myopia progression as much when compared with lesser amounts of myopic peripheral defocus. A study imposing +4.00 D or −4.00 D lenses in guinea pigs found that myopia progression and axial elongation were enhanced when superimposing a +4.00 D peripheral myopic defocus lens [38]. This suggested that the local retinal area can decode whether it is a clear or blurred signal. If the defocus is above the threshold of signal detection, this function will fail, and might lead to myopia progression [38]. The depth of focus (DOF), which could represent the threshold of blur detection, has been investigated in a human study. It was reported that DOF increased with eccentricity [40] and DOF could reach ±6 D in the mid-periphery [41], which suggested that the amount of myopic defocus in myopia control lenses may need to be varied across the retinal eccentricities to maintain a myopic defocus image shell. 

The myopic children in the current study showed a large variation in RPR, from −1.25 D to as much as +4.00 D, at the 10 N and 20 N retina. Those children in the DIMS group who had baseline myopic RPR might be considered to have received too much myopic defocus at mid-periphery and failed to benefit from the myopic defocus signal. For a better myopia control efficacy, further modification of DIMS lenses may point to different dosage myopic defocus (such as, +1.50 D and +2.50 D) for the children with baseline myopic RPR or less hyperopic RPR.

Notably, the relationship that baseline RPR influenced myopia control effects was only found within 20° of the nasal retina. There have been suggestions that the nasal retina is more sensitive to defocus signals to slow eye growth [9,42], and one study reported that the influence of myopic defocus on refractive development is reduced with increasing eccentricity [43]. Therefore, mapping the peripheral retinal profile, or at least the nasal near-peripheral retina, for customising the required defocus and avoiding producing a strong peripheral myopic defocus could be vital for optimising myopia control effects. 

Although the baseline RPR in the DIMS group can only explain 15% to 20% of myopia progression and axial elongation according to the multilinear analysis, other factors such as incorrect centration of the pupil centre and eye-foveal axis might influence the actual peripheral defocus exposure and final myopia control effects. Another possible factor, which has not been considered in this study, was the hyperopic retinal blur due to the lag of accommodation during near work, which might also contribute to the summation of defocus power and affect the final myopic defocus effectiveness [30]. Further studies to incorporate individual lag of accommodation for resultant myopic defocus analysis would provide additional information and understanding.

The current study described the RPR profile in myopic schoolchildren 8 to 13 years old, and reported the role of RPR in myopia control. Peripheral refraction can be measured rapidly in clinical settings, and the RPR can be calculated easily by clinicians. Thus, RPR may provide a clinically useful measure for optimising and monitoring myopia control. 

## 5. Conclusions

The DIMS lens provides effective myopia control and is more effective in myopia control for children with baseline hyperopic RPR than for children with myopic RPR, and this may partially explain why myopia control effects vary among myopic children. Customised myopic defocus for individual subjects may optimise myopia control effects, and further research to investigate the optimal dosage, with consideration of peripheral retinal profile, is warranted.

## Figures and Tables

**Table 1:** Multiple linear regressions between relative peripheral refraction and myopia progression, axial elongation with relative peripheral refraction as the independent variable.

Relative Peripheral Refraction at Baseline	Myopia Progression				Axial Elongation				
	Regression Coefficient	95% CI for B		*p* ^†^	Regression Coefficient		95% CI for B		*p* ^†^
	B	Lower Bound	Upper Bound		B		Lower Bound	Upper Bound	
Adjusting for Age, Gender and Initial Refractive Error					Adjusting for Age, Gender and Initial Axial Length				
***DIMS group***									
10 T	0.00	−0.24	0.24	0.99	0.03		−0.08	0.11	0.74
20 T	−0.08	−0.17	0.08	0.45	0.03		−0.04	0.05	0.77
30 T	0.03	−0.08	0.10	0.81	−0.08		−0.05	0.02	0.53
10 N	0.36	0.19	0.74	0.001	−0.35		−0.29	−0.08	0.001
20 N	0.35	0.08	0.33	0.001	−0.30		−0.12	−0.02	0.004
30 N	0.25	0.01	0.20	0.03	−0.22		−0.08	−0.002	0.05
***SV group***									
10 T	0.06	−0.28	0.48	0.59		0.03	−0.12	0.15	0.77
20 T	−0.06	−0.18	0.11	0.62		0.11	−0.02	0.08	0.29
30 T	−0.07	−0.13	0.08	0.65		0.14	−0.02	0.06	0.29
10 N	0.37	0.23	0.90	0.001		−0.15	−0.22	0.03	0.13
20 N	0.36	0.12	0.49	0.001		−0.23	−0.14	−0.01	0.02
30 N	0.35	0.07	0.29	0.002		−0.24	−0.09	0.009	0.02

^†^*p* < 0.008 was considered as the statistical significance.

**Table 2:** The difference in myopia progression and axial elongation between children with myopic RPR and hyperopic RPR at 10 N in the DIMS and SV group, respectively.

	Myopic RPR at 10 N		Hyperopic RPR at 10 N		Mean Difference	^†^*p* Value
	Mean ± SD	n	Mean ± SD	n		
***DIMS group***						
Myopia progression (D)	−0.61 ± 0.60	27	−0.25 ± 0.44	52	−0.36 ± 0.14	0.009
Axial elongation (mm)	0.32 ± 0.24	27	0.16 ± 0.18	52	0.16 ± 0.05	0.001
***SV group***						
Myopia progression (D)	−1.10 ± 0.58	27	−0.84 ± 0.59	54	0.26 ± 0.14	0.06
Axial elongation (mm)	0.55 ± 0.27	27	0.51 ± 0.22	54	0.04 ± 0.05	0.48

^†^*p* < 0.05 was considered as the statistical significance.

**Table 3:** The difference in myopia progression and axial elongation between children with myopic RPR and hyperopic RPR at 20 N in the DIMS and SV group, respectively.

	Myopic RPR at 20 N		Hyperopic RPR at 20 N		Mean Difference	^†^*p* Value
	Mean ± SD	n	Mean ± SD	n		
***DIMS group***						
Myopia progression (D)	−0.72 ± 0.64	12	−0.31 ± 0.48	67	−0.40 ± 0.16	0.01
Axial elongation (mm)	0.34 ± 0.24	12	0.19 ± 0.20	67	0.15 ± 0.07	0.02
***SV group***						
Myopia progression (D)	−1.14 ± 0.53	11	−0.89 ± 0.60	70	−0.25 ± 0.20	0.19
Axial elongation (mm)	0.60 ± 0.28	11	0.51 ± 0.23	70	0.08 ± 0.08	0.27

^†^*p* < 0.05 was considered as the statistical significance.

## Data Availability

The data presented in this study are available on request from the corresponding author.
